# Sequence, characterization and pharmacological analyses of the adipokinetic hormone receptor in the stick insect, *Carausius morosus*

**DOI:** 10.3389/fendo.2025.1601334

**Published:** 2025-07-17

**Authors:** Gerd Gäde, Jinghan Tan, Salwa Afifi, Jean-Paul V. Paluzzi, Graham E. Jackson, Heather G. Marco

**Affiliations:** ^1^ Department of Biological Sciences, University of Cape Town, Rondebosch, South Africa; ^2^ Department of Biology, York University, Toronto, ON, Canada; ^3^ Department of Chemistry, University of Cape Town, Rondebosch, South Africa

**Keywords:** adipokinetic hormone receptor, G protein-coupled receptor, *in vitro* receptor activation, structure-activity relationship, alanine replacement series, stick insect, metabolism, pharmacological analyses

## Abstract

**Background:**

Adipokinetic/hypertrehalosaemic hormone (AKH/HrTH), corazonin (Crz) and the AKH/Crz-related peptide (ACP) are neuropeptides considered homologous to the vertebrate gonadotropin-releasing hormone (GnRH). AKH/HrTH are important peptidergic metabolic regulators in insects that are crucial to provide energy during periods of high output mobility or when large amounts of energy-rich substrates are synthesized (for example, during vitellogenesis). AKH functions via a G protein-coupled receptor. Understanding which residue of the peptide (the ligand), activates the receptor with high efficacy is an important step to get insights into the ligand-receptor interaction, which is essential for further research on creating a model of how the ligand behaves in the binding pocket of the receptor. Such data are necessary for the search of non-peptidic mimetic agonists or antagonists in pesticide design.

**Methods:**

Using bioinformatics and cloning techniques, the complete coding sequence of an AKH receptor was cloned and sequenced from fat body tissues and nervous tissues from the Indian stick insect, *Carausius morosus*. The resulting Carmo-AKHR was then expressed in a mammalian cell line where it could couple with a Gq protein to mediate calcium mobilization *in vitro* and cause bioluminescence when activated by a ligand. This receptor assay was used not only with the natural AKH ligands of the stick insect, but also with AKHs from other species and analogs with targeted modifications. A phylogenetic analysis of Carmo-AKHR with the AKH receptors and related receptors from other insects was also carried out.

**Results:**

The stick insect AKH receptor was successfully cloned and sequenced from fat body and, separately, from nervous tissues. Comparison with known insect AKH, Crz and ACP receptors clearly put the stick insect receptor in the AKH clade and as sister group to other putative Phasmatodean AKH receptors. Moreover, the receptor expressed in mammalian cells was only activated by AKH and not by Crz or ACP indicating a true AKH receptor. Structure-activity studies in an Ala replacement series revealed the ligand residues that are absolutely essential for activating the AKHR: the N-terminal pGlu, Phe^4^, Trp^8^ and the C-terminal carboxyamide. Almost as important are Thr^3^ and Thr^5^ since their replacement reduced the efficacy more than a 100-fold, whereas Thr^10^ can be replaced without any real loss of activity. When substituted by Ala at positions 2, 6, 7 and 9, the ligand is somewhat affected with the loss of receptor activation being between 5- to 20-fold. Chain length of the ligand is important for the receptor: an octa- or nonapeptide with the same sequence otherwise as the endogenous stick insect ligand, display a 5- to 10 fold reduced activity. Carefully selected naturally occurring AKH analogs from other insects support the above results.

**Conclusions:**

The AKH receptor from stick insects (Phasmatodea) cluster together in one clade distinct from other insect AKHRs, although still similar enough to be an insect AKHR, as opposed to the other GnRH-related receptors of insects, such as ACP and Crz receptors. The phylogenetic analyses support the data obtained from other studies involving receptors for AKH, Crz and ACP peptides. The receptor assay results with AKH analogs corroborated most of the results obtained previously using *in vivo* studies, thus emphasizing that the endogenous AKHs operate through this receptor to cause hypertrehalosemia in the stick insect. It is also clear that certain residues of the AKH peptides are consistently important in their interaction with the cognate AKH receptor, while other amino acid residues are of different importance to AKH receptors on a broad species- or group-specific manner. The previously observed peculiarity that hypertrehalosemia, in response to AKH injection, is only measurable in stick insects ligated below the head is discussed. No explanations for this, however, can be inferred from the current study.

## 1 Introduction

The adipokinetic hormone (AKH) family consists of short peptides with characteristic features that regulates intermediary metabolism in insects; these bioactive peptides have therefore, also been named according to the chief metabolite that is mobilized into the insect hemolymph, viz. diacylglycerides, trehalose and proline, thus labeled as AKH, hypertrehalosemic hormone (HrTH) and hyperprolinemic hormone, respectively. Other insect AKHs are named after the site of synthesis, i.e. the corpora cardiaca (CC), while a few are named after other biological actions, such as cardioacceleratory hormone (CAH; increasing the heart rate) or red pigment-concentrating hormone (RPCH; the action of these hormones in decapod crustaceans). In this publication, we will mostly use the generic term “AKH” for the peptide and in reference to the cognate G protein-coupled receptor, “AKHR”. To effect a metabolic action in insects, the AKH peptides act via the AKHR on storage organs/tissues such as the fat body to release metabolites into circulation. Because of structural relatedness, AKHs and their receptors are part of a large superfamily that include the vertebrate gonadotropin releasing hormone (GnRH), corazonin (Crz) and the adipokinetic-corazonin-related peptide (ACP), along with the respective receptors (see 1).

The study animal of the current paper, the Indian stick insect *Carausius morosus*, has fascinated scientists and lay-persons alike for many years: from its curious body form that mimics twigs (sticks), to its predator avoidance strategy of playing dead (thanatosis), and its reproductive strategy (“virgin birth”; parthenogenesis). *C. morosus* has become synonymous with scientific studies on the control of locomotion (eg. 2–4), while from as early as 1979, intriguing results emanated from metabolic investigations with *C. morosus*: it was found that an extract of the CC had hypertrehalosemic activity in cockroaches, hyperlipemic activity in locusts and glycogen phosphorylase was activated in the fat body of locusts, but the CC extract was not active in the stick insect itself (5, 6) unless a ligature was applied between the head and the thorax of the stick insects prior to injection of the AKH test material (7). In such ligatured *C. morosus* specimens, a small but statistically significant increase in carbohydrate concentration was measured in the hemolymph after injection of conspecific CC material, thus a hypertrehalosemic effect (7). The biological effect was ascribed to two peptides that were isolated from the CC via reverse-phase-high performance liquid chromatography (RP-HPLC) and named Carmo-HrTH-I and -II in the order of elution (8, 9). The primary structure of these entities was elucidated by fast atom bombardment mass spectrometry and nuclear magnetic resonance (NMR) spectroscopy as a decapeptide in each case, identical except that Carmo-HrTH-I boasts an unusually modified tryptophan residue, viz. a C-bonded alpha- mannopyranose (10–12). Recently a method was developed to synthesize such C-mannosylated Trp constructs, and Carmo-HrTH-I was successfully synthesized and validated as having the correct chromatographic and physical parameters (13) and the same biological activity as the natural peptide (14). Up to then, physiological studies on the Indian stick insect was performed with synthetic Carmo-HrTH-II and to a limited extent with natural Carmo-HrTH-I, for example on the effect on heart rate (15) and on metabolism (16). From these studies, it was apparent that the AKH peptides from *C. morosus* were equally effective biologically. Other stick insect species were also examined in physiological studies (17) but knowledge on AKH peptide-receptor interaction in phasmids remain scarce. In 2020 indirect biological assays at organismal level were carried out with ligated *C. morosus* specimens to assess AKHR-ligand responses by measuring the metabolic output after injection of strategically modified Carmo-HrTH peptide analogs and AKH bioanalogs found in other insect species (16). Such *in vivo* structure-activity relationship (SAR) studies presented a good picture of which amino acids in the decapeptide molecule may be important during receptor interaction but there is the caveat that there are many steps between the initial point of ligand-receptor binding and the final measured output of a change in circulating carbohydrate concentration. A more direct way to study ligand-receptor interactions would be to clone the receptor and express it in a cell line with specific constructs that enable the real-time monitoring of calcium release upon ligand-receptor binding via a bioluminescence signal; such a heterologous *in vitro* assay system was successfully implemented for SAR investigations on the AKHR of *Drosophila melanogaster*, *Anopheles gambiae*, *Glossina morsitans* and *Aedes aegypti* (18–20).

In the current study, the same approach is followed to clone the AKHR from *C. morosus*, to biologically validate it as Carmo-AKHR through *in vitro* receptor expression assays and to confirm insights gleaned from *in vivo* SAR studies (16) into the importance of the termini and side chains of the amino acids of Carmo-HrTH-II for interacting with the AKHR. A partial amino acid sequence of the purported *C. morosus* AKHR was reported from a transcriptome and used in *in silico* modeling (21) but this receptor was never cloned or biologically validated; moreover, the full sequence and the methodology was never deposited or published for full scrutiny and the C-terminal amino acids differed vastly from a shorter sequence published in a 2021 study also from a transcriptome and not characterized as an AKHR (3). Hence, the current study presents the fully characterized receptor sequence in the Indian stick insect for the first time, and it is hoped that this information may aid in clarifying the observed lack of AKH function in the stick insect when the circulatory system from the head is not separated from the rest of the body. Furthermore, the cloned Carmo-AKHR sequence can be used as template to build an independent model for studying molecular interactions of the AKHR and the endogenous ligands, as well as for screening compound libraries to identify potential agonists or antagonists that may interfere with biological action. Such a rapid *in silico* screening strategy can assist with the identification of lead substances for the development of a pest insect-specific ‘green insecticide” that interacts with the AKHR of the pest insect only, which would also be worthwhile for stick insects as they occasionally achieve pest status (22).

## 2 Materials and methods

### 2.1 Insects and natural peptide extraction

Adult *C. morosus* were collected from ivy hedges in the southern suburbs of Cape Town and kept in large cages at the Department of Biological Sciences. They were fed twice a week with fresh ivy leaves in jars of water.

The corpora cardiaca (CC) were dissected from 10 stick insects into 80% methanol with the help of a stereo microscope (20- fold magnification) and the contents extracted by sonication for 20 s. After centrifugation, the CC extracts were dried in a vacuum concentrator and subsequently subjected to RP-HPLC to isolate, collect and quantify the two endogenous AKH peptides, Carmo-HrTH-I and -II, as previously described (9, 14, 16).

### 2.2 The stick insect AKH receptor and the preparation of its expression construct

*C. morosus* adult tissue samples (n=8) were dissected in nuclease-free phosphate-buffered saline (PBS); fat body tissues and neural tissues (brain, corpus cardiacum/corpus allatum, and ventral nerve cord were pooled) were stored in RNAlater (ThermoFisher Scientific, Burlington, ON) to preserve RNA integrity and total RNA was later isolated as described (23) and quantified using UV spectrophotometry using a Synergy Multi-Mode Microplate Reader (BioTek, Winooski, VT, USA). First-strand cDNA synthesis was completed with 1 µg total RNA as template using the iScript reverse transcription supermix (Bio-Rad, Mississauga, ON) that includes a blend of oligo(dT) and random primers providing an unbiased synthesis of target genes, including both 5’ and 3’ regions. Reactions included a 5 min priming step at 25°C followed by reverse transcription for 60 min at 46°C and finally a RT inactivation step for 1 min at 95°C. After synthesis, cDNA was diluted 10-fold with nuclease-free double-distilled water prior to use as a template in PCR.

In a first step of primer design, Phasmid genome datasets available at the time, including *Clitarchus hookeri* (24) and *Medauroidea extradentata* (25), were screened via local tblastn blast analysis using Geneious Prime bioinformatics software (Biomatters Ltd., Auckland, New Zealand) and the desert locust AKHR (26) as a protein query. Based on the highest scoring matches, a series of degenerate primers were designed over regions with strong similarities. The primer pair Cm85F (5’-GTCGGYAACATCACCGT-3’) and Cm742R (5’-GATGGTCATCTTGAGAGTGC-3’) were used to amplify a partial sequence; PCR amplification involved using Q5 High Fidelity DNA Polymerase (New England Biolabs, Whitby, ON) with a 0.5 μM primer concentration and following manufacturer recommended cycling conditions including initial denaturation (98°C) for 30 s, 35 cycles of denaturation (98°C) for 5 s, annealing (62°C) for 20 s and extension (72°C) for 30 s, and a final extension (72°C) for 2 min. The amplicon was purified using a PCR purification kit (GeneBio Inc., Burlington, ON) and target specificity was verified by Sanger sequencing (Centre for Applied Genomics, Hospital for Sick Children, Toronto, ON).

In a second step, primers were designed at the predicted start (ATGAGCACGCGTGCAGCTGG) and stop (TCACCTGGCTGTGGGCGTGT) codons of two partial putative AKH receptor sequences [Accession numbers: MT879330, MT879331, MT879332) reported in (3)]. The presumed complete ORF was cloned separately from fat body and neural tissue cDNA. PCR amplification conditions followed those described above with the following changes, the annealing temperature used was 72°C and extension step during cycling was increased to 90 s due to the larger amplicon size. This amplified ORF product was verified by Sanger sequencing (Centre for Applied Genomics, Hospital for Sick Children, Toronto, ON) to confirm base accuracy matched the two earlier reported isoforms (3). The receptor structures were visualized using the open-source tool, Protter (27). These cloned receptor isoforms were then used to incorporate a Kozak translation initiation sequence and restriction sites (5’ Hind*III* and 3’ Xba*I*) for directional cloning in pcDNA3.1^+^ and pBudCE4.1 mammalian expression vectors as described previously (20).

In a third step of primer design to amplify the complete ORF of the *C. morosus* AKHR, personal communication was initiated with the research group leader who had reported a longer AKHR sequence from the stick insect but without public deposition of the sequence (21). Based on the shared information, primer pairs were designed outside of the predicted ORF, including portions of the 5’ and 3’ untranslated region (UTR) of the nucleotide sequence: a sense primer (5’-GTGATAAGGGAGGCTGTCG -3’) positioned 75 bp upstream of the predicted start codon and an anti-sense primer (5’-GGTGCAGGACATGCCTCTGG -3’) located 31 bp downstream of the predicted stop codon. For PCR, Q5 high-fidelity polymerase (New England Biolabs, Whitby, ON) was used following the recommended guidelines due to its superior performance including ultra-low error rates. PCR amplification conditions were identical to those described above, but with an annealing temperature of 66°C and extension step during cycling increased to 120 s due to the larger amplicon size. This larger amplicon was confirmed by Sanger sequencing (Centre for Applied Genomics, Hospital for Sick Children, Toronto, ON) and subcloned into mammalian expression vectors as described above.

To ensure optimal expression in the heterologous cell assay, the receptor construct was synthesized by a contract research organization (Genscript USA Inc., Piscataway, NJ) in pcDNA3.1^+^ using codon optimization for mammalian expression and this sequence was flanked by a ribosomal skipping peptide (28) allowing co-expression of eGFP to monitor transfection efficiency and to streamline selection of a stable cell line expressing the *C. morosus* AKH receptor.

### 2.3 Stable cell line generation for the calcium bioluminescence reporter assay

Functional activation of Carmo-AKHR was carried out by following mammalian cell culture procedures as previously described (20, 29) with the following modifications. Pharmacological analyses were performed in Chinese hamster ovary (CHO)-K1 cells transfected with linearized pcDNA-Carmo-AKHR-P2A-eGFP using Lipofectamine LTX with Plus Reagent (Invitrogen, Burlington, ON) and following the manufacturer’s recommended DNA to transfection reagent ratios. At 24 h post-transfection, cells were selected in DMEM:F12 media containing 10% heat-inactivated fetal bovine serum (Wisent, St. Bruno, QC), 1x antimycotic-antibiotic and 600 µg/mL geneticin (G418) until clonal recombinant cells were evident in transfected cells while no live cells were present in non-transfected CHO-K1 cells (control). Individual clonal recombinant colonies were seeded into separate wells of a 96 well cell-culture dish (Sarstedt Inc., Montreal, QC) and grown to confluence while maintaining geneticin selection. To identify clonal recombinant lines with optimal Carmo-AKHR activity yielding the greatest signal-to-noise ratio, individual clonal recombinant lines were assayed in the bioluminescence reporter assay as described previously (30). Briefly, the Carmo-AKHR recombinant clonal lines were transfected (as outlined above) with a pcDNA3.1+ plasmid encoding the calcium-activated photoprotein, aequorin (31). At 48 h post transfection with aequorin, Carmo-AKHR recombinant cells were prepared as previously described (31) for the bioluminescent reporter assay with receptor activation tested using synthetic *A. aegypti* AKH (32) and CCHamide1 (33), the latter serving as a negative control. The recombinant cell line showing the optimal luminescent response with greatest signal-to-noise ratio was expanded for preparation of cryo-stocks and subsequent detailed investigation of receptor specificity.

The CHO-K1 recombinant cell line stably expressing Carmo-AKHR was maintained in DMEM:F12 media containing 10% heat-inactivated fetal bovine serum (Wisent, St. Bruno, QC), 250 μg/mL Geneticin (MilliporeSigma Canada Ltd., Oakville, ON) and 1X antimycotic-antibiotic (ThermoFisher Scientific, Whitby, ON). In preparation for large-scale bioluminescent receptor assay, cells were seeded into multiple T75 cell culture-treated flasks (GeneBio Inc., Burlington, ON) at approximately 90% confluency, and allowed to recover for several hours prior to transfection with pcDNA3.1+ plasmid encoding aequorin. Transfection was completed with PolyJet™ DNA *In Vitro* Transfection Reagent (FroggaBio, Toronto, ON) following manufacturer recommendations for transfection of adherent cells. At 48 hours post-transfection, cells were detached from culture flasks using Dulbecco’s PBS (Wisent, St. Bruno, QC) containing 5 mM Ethylenediaminetetraacetic acid (EDTA) and were prepared for the functional assay as previously described (30).

Synthetic peptide stocks of naturally-occurring AKH-related peptides [corazonin, adipokinetic hormone corazonin-like peptide (ACP)], alanine-substituted analogs of Carmo-HrTH-II, natural *C. morosus* AKH peptides, and several AKHs from other arthropod species in synthetic form were used for preparation of serial dilutions all of which were prepared in assay media (DMEM-F12 media containing 0.1% bovine serum albumin, 1X antimycotic-antibiotic) and loaded in triplicate or quadruplicate into 96-well white luminescence plates (Greiner Bio-One, Germany). Cells stably expressing Carmo-AKHR and transiently expressing aequorin were loaded with an automated injector unit into each individual well containing different test compounds at various concentrations, including assay media alone (negative control wells) and 50 μM ATP (positive control wells) acting on endogenously expressed purinoceptors (34, 35). Immediately after injection of the cells into each well, luminescence was measured for 20 s using a Synergy 2 Multi-Mode Microplate Reader (BioTek, Winooski, VT, USA). Calculations, including determination of EC_50_ values, were conducted in GraphPad Prism 10.4.1 (GraphPad Software, San Diego, USA) from dose-response curves from 3 to 4 independent biological replicates. Data were analyzed using a one-way or two-way ANOVA and Tukey’s multiple comparisons *post-hoc* test, with differences between treatments considered significant if p<0.05.

### 2.4 Sequence analysis of the complete *C. morosus* AKH receptor

The deduced protein sequence from the cloned Carmo-AKHR sequence was used to identify the hydrophobic transmembrane helical domains of the receptor employing the Constrained Consensus TOPology (CCTOP) prediction server (36). In addition, potential N-linked glycosylation sites on the N-terminal or extracellular domains of the Carmo-AKH receptor were identified using the NetNGlyc 1.0 server (https://services.healthtech.dtu.dk/services/NetNGlyc-1.0/) (37), while potential phosphorylation sites on intracellular loops and the C-terminal cytoplasmic tail were predicted using the NetPhos 3.1 server (https://services.healthtech.dtu.dk/services/NetPhos-3.1/) (38, 39). Finally, the predicted post-translational modifications along with proteoforms were visualized using the open-source tool, Protter (27).

### 2.5 Phylogenetic analysis of Carmo-AKHR

The deduced Carmo-AKHR protein sequence was aligned to AKH-, ACP-, and Crz receptors from other insect species, along with the human gonadotropin-releasing hormone receptor 1, using ClustalW in MEGA11: Molecular Evolutionary Genetics Analysis version 11 (40). The species information and sequence accession numbers are provided in the figure. Relationships between the various receptor sequences were determined through maximum-likelihood phylogenetic analysis methods (41). The reliability of the inferred tree was assessed by bootstrap analysis using 1000 replicates (42).

### 2.6 Synthetic peptides

All synthetic peptides were initially prepared as stock solutions at a concentration of 1mM in dimethyl sulfoxide and subsequently diluted to the desired concentration in BSA assay buffer. For primary structures of AKH peptides and analogs see **Tables 1**, **2**.

**Table 1:** Analogs of Carmo-HrTH-II tested on the *Carausius morosus* AKH receptor in *in vitro* bioluminescence assays and the resulting EC_50_ values.

Peptide/analog name	Peptide sequence ^a^	EC_50_ value (M) ^b^	Fold- reduction in activity ^c^
Carmo-HrTH-II	pELTFTPNWGT amide	7.36E-8	–
Carmo-HrTH-I	pELTFTPN**W^(d)^** GT amide	8.97E-8	1.22
Carmo-HrTH-II free acid	pELTFTPNWG**T-OH**	ND*	>1360
Carmo-HrTH-II minus pGlu	**L**TFTPNWGT amide	ND*	>1360
[N-Ac. Ala]^1^-analog	**^(e)^A**LTFTPNWGT amide	ND*	>1360
Ala^2^-analog	pE**A**TFTPNWGT amide	6.93E-7	9.42
Ala^3^-analog	pEL**A**FTPNWGT amide	7.69E-5	1045.82
Ala^4^-analog	pELT**A**TPNWGT amide	ND	>1360
Ala^5^-analog	pELTF**A**PNWGT amide	8.58E-5	1166.83
Ala^6^-analog	pELTFT**A**NWGT amide	4.04E-7	5.49
Ala^7^-analog	pELTFTP**A**WGT amide	3.10E-7	4.21
Ala^8^-analog	pELTFTPN**A**GT amide	ND*	>1360
Ala^9^-analog	pELTFTPNW**A**T amide	1.48E-6	20.18
Ala^10^-analog	pELTFTPNWG**A** amide	1.10E-8	0.15

^a^Amino acid residues in bold text indicate a substitution relative to Carmo-HrTH-II.

^b^EC_50_ values were calculated from dose-response activation curves.

^c^The fold reduction in receptor activation relative to Carmo-HrTH-II.

^d^The sequence of Carmo-HrTH-I: pELTFTPNW^(C-mannosylated)^GT amide

^e^pGlu^1^ is replaced by [N-acetylated Alanine]

*denotes EC_50_ not determined (ND) as peptide/analog had minimal activity up to the highest concentration tested.

**Table 2:** AKH peptides tested on the Carmo-AKHR in *in vitro* bioluminescence assays.

Peptide name	Native source	Peptide sequence ^a^	EC_50_ value (M) ^b^	Fold-reduction in activity ^c^
Carmo-HrTH-II	*Carausius morosus*	pELTFTPNWGT amide	7.36E-08	–
Triin-AKH	*Triatoma infestans*	pELTFTPNWG**–** amide	5.48E-07	7.45
Peram-CAH-II	*Periplaneta americana*	pELTFTPNW**––** amide	3.88E-07	5.28
Phyle-CC	*Phymateus leprosus*	pELTFTPNWG**S** amide	1.80E-08	0.24
Locmi-AKH-I	*Locusta migratoria*	pEL**N**FTPNWGT amide	4.14E-06	56.30
Phymo-AKH	*Phymateus morbillosus*	pEL**N**FTPNWG**S** amide	5.05E-06	68.66
Polae-HrTH	*Polyphaga aegyptiaca*	pE**I**TFTPNW**––** amide	8.76E-06	119.12
Aedae-AKH	*Aedes aegypti*	pELTFTP**S**W**––** amide	1.03E-06	14.00
Peram-CAH-I	*Periplaneta americana*	pE**VN**F**S**PNW**––** amide	ND*	>1360
Panbo-RPCH	*Pandalus borealis*	pEL**N**F**S**P**G**W**––** amide	3.35E-05	455.74

^a^Amino acid residues in bold text indicate a substitution relative to Carmo-HrTH-II.

^b^EC_50_ values were calculated from dose-response activation curves.

^c^The fold reduction in receptor activation relative to Carmo-HrTH-II.

*denotes EC_50_ not determined (ND) as peptide/analog had minimal activity up to the highest concentration tested.

-indicates the absence of an amino acid relative to Carmo-HrTH-II, as in the case of nona- and octapeptides.

Peptide analogs with a single amino acid substituted by an alanine residue at each position of the decapeptide Carmo-HrTH-II were synthesized by Pepmic Co., Ltd. (Suzhou, China) at a purity of at least 85%. Carmo-HrTH-II with modified termini (a free acid, as well as a nonapeptide representing Carmo-HrTH-II minus the N-terminal pGlu) were custom-synthesized (at least 85% purity) by Dr. S. Kyin (Biotechnology Centre, University of Illinois, Urbana-Champaign, USA).

Peptides (at least 95% purity) were purchased from Synpeptide Co., Ltd (Shanghai, China): the decapeptides Locmi-AKH-I, Phymo-AKH, and Phyle-CC; the nonapeptide Triin-AKH; and the octapeptides Panbo-RPCH, Peram-CAH-I, Peram-CAH-II, and Polae-HrTH. The GnRH-related peptides of the mosquito *Aedes aegypti*, including Aedae-AKH, Aedae-Crz (pETFQYSRGWTN amide) and Aedae-ACP (pEVTFSRDWNA amide) were commercially synthesized (Genscript Inc., Piscataway, NJ) and stocks prepared as described previously (30, 32).

## 3 Results

### 3.1 Cloning and validation of Carmo-AKHR; receptor features

The first attempt at cloning an AKHR from the Indian stick insect was in the absence of a genome or published transcriptome. A 615 bp amplicon was obtained with PCR and degenerate primers based on a consensus sequence from scanning the available genomes of other Phasmids. This amplicon yielded a deduced protein sequence of 205 amino acid residues, corresponding to a region spanning the predicted C- and N-terminal regions of the first and sixth transmembrane domains, respectively (**Supplementary Figure S1**). To amplify the complete ORF, primers were designed from sequence information published in 2021 deduced from a *C. morosus* transcriptome (3). The resulting amplicons were sequenced and represented two isoforms of a receptor, matching the published sequences. The seemingly complete receptor sequences included seven predicted transmembrane domains, as well as an extracellular N-terminus and intracellular C-terminus (see **Supplementary Figure S2**) as visualized with the open-source tool, Protter (27). The predicted protein translation from these clones yielded deduced proteins of 401 (variant #1) or 403 (variant #2) residues. These receptor isoforms were directionally cloned into mammalian expression vectors and expressed in CHO-K1 cell on its own (using pcDNA3.1^+^), or together with murine promiscuous G protein (using pBudCE4.1) for testing with synthetic insect AKH-related peptides, as described (20, 32) to verify if any functional differences were evident between the two receptor variants or based on the expression construct. None of the receptor constructs exhibited any functional activation responses, neither with nor without the promiscuous G protein (data not shown). This led to the conclusion that the amplified clones might not reflect the complete receptor protein sequence after all, especially considering a 2020 study that alluded to an unpublished predicted 528 amino acid long *C. morosus* AKHR from transcriptomic data (21).

**Figure 1:**
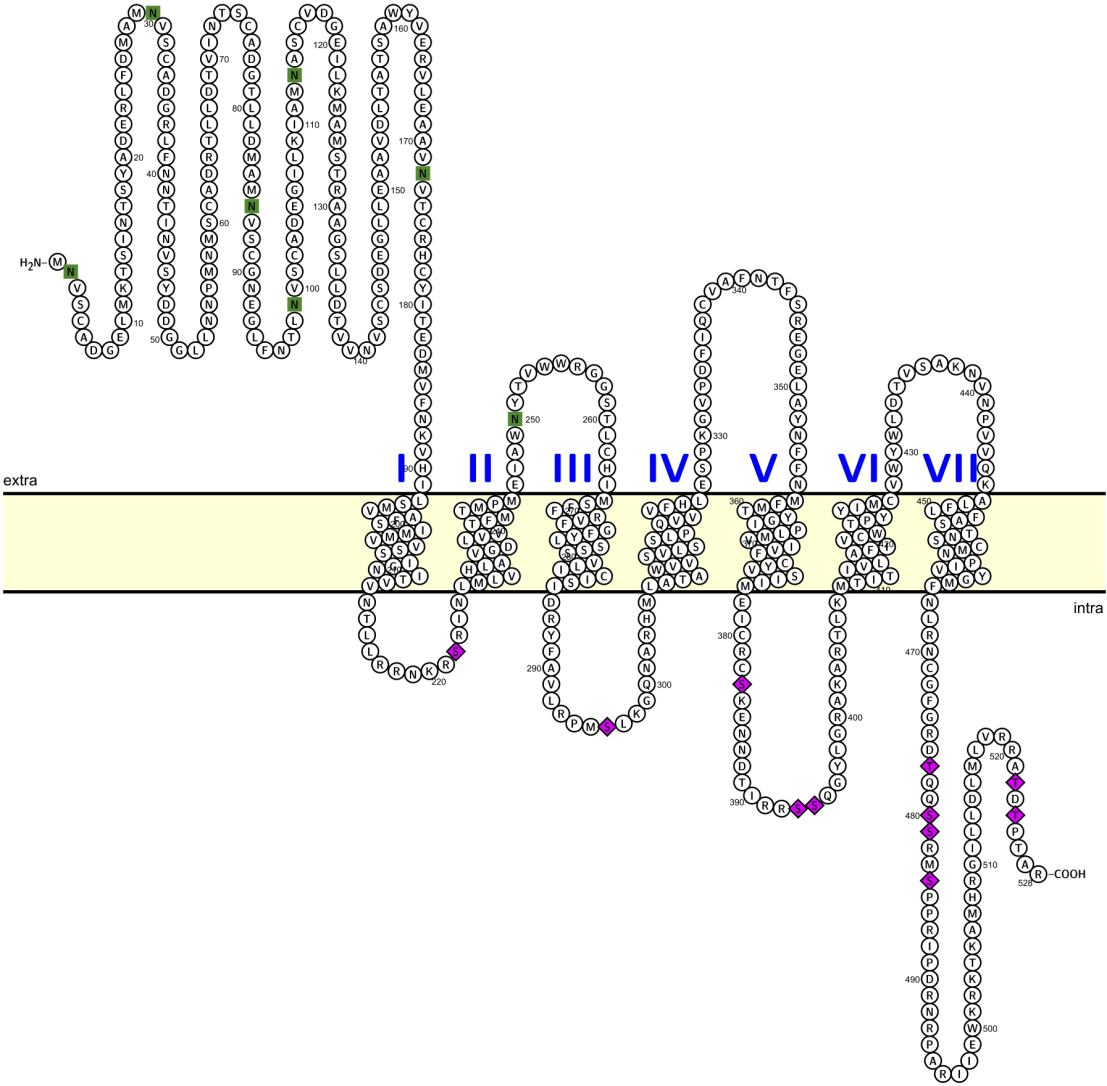
Predicted topology and post-translational modifications of the complete and functional *C. morosus* AKH receptor. The deduced 528 amino acid Carmo-AKH receptor protein sequence was used to predict membrane topology (residues in membrane), N-linked glycosylation sites (residues denoted by green squares) along with potential phosphorylation sites within cytoplasmic domains of the receptor (residues denoted by purple diamonds). See the methods section for full description of steps in the analysis and prediction of post-translational modifications along with references for the web-based applications. Protter, an open-source tool, was used prepare the schematic of the receptor topology as well as label the predicted post-translational modifications.

The third attempt to clone the AKHR (after consultation with the lead researcher (see 21), resulted in a full and functional AKHR. With the latest primer set, a sense primer positioned 75 bp upstream of the predicted start codon and an anti-sense primer located 31 bp downstream of the predicted stop codon yielded a similar ~1.7kb sized product using either fat body or nervous system cDNA as template. Amplicons from fat body and nervous tissue were processed separately to enable identification of any splice variants or isoforms like those previously sequenced as shorter, non-functional receptor clones (see above; **Supplementary Figure S2**). Based on Sanger sequencing of this new amplified product (of 1693 bp), only a single amplicon was identified (GenBank accession: PV125520) with no nucleotide differences irrespective of the template (fat body or nervous tissue) from which it had been independently amplified. Within this larger amplicon that contained portions of both the 5’ and 3’ UTRs, was a 1587 bp ORF that yielded a deduced protein sequence of 528 amino acids (**Figure 1**) with an N-terminal extension of 125 residues compared to the deduced protein sequence from earlier amplified clones (see **Supplementary Figure S2**). A schematic overview is provided (**Supplementary Figure S3**) to illustrate and describe the different pathways followed to result in the successful cloning of the full length (complete) Carmo-AKHr; functional validation was demonstrated through specific receptor activation by AKH peptides only.

The complete *C. morosus* AKH receptor shares various features with members of the rhodopsin GPCR superfamily, including seven hydrophobic transmembrane domains, an extracellular N-terminus and intracellular C-terminus. With respect to post-translational modifications, our analysis predicts several N-linked glycosylation sites within the N-terminal extracellular domain as well as a single site within the first extracellular loop located between the second and third transmembrane domains (**Figure 1**), which serve as important sorting signals for directing the receptor to the plasma membrane (43). Notably, the sequence analysis of CarmoAKHr also identified several predicted phosphorylation sites within each of the intracellular loops along with the C-terminal cytoplasmic tail (**Figure 1**), suggesting such sites might play important roles in the signaling properties of this receptor.

### 3.2 Phylogenetic analyses

**Figure 2:**
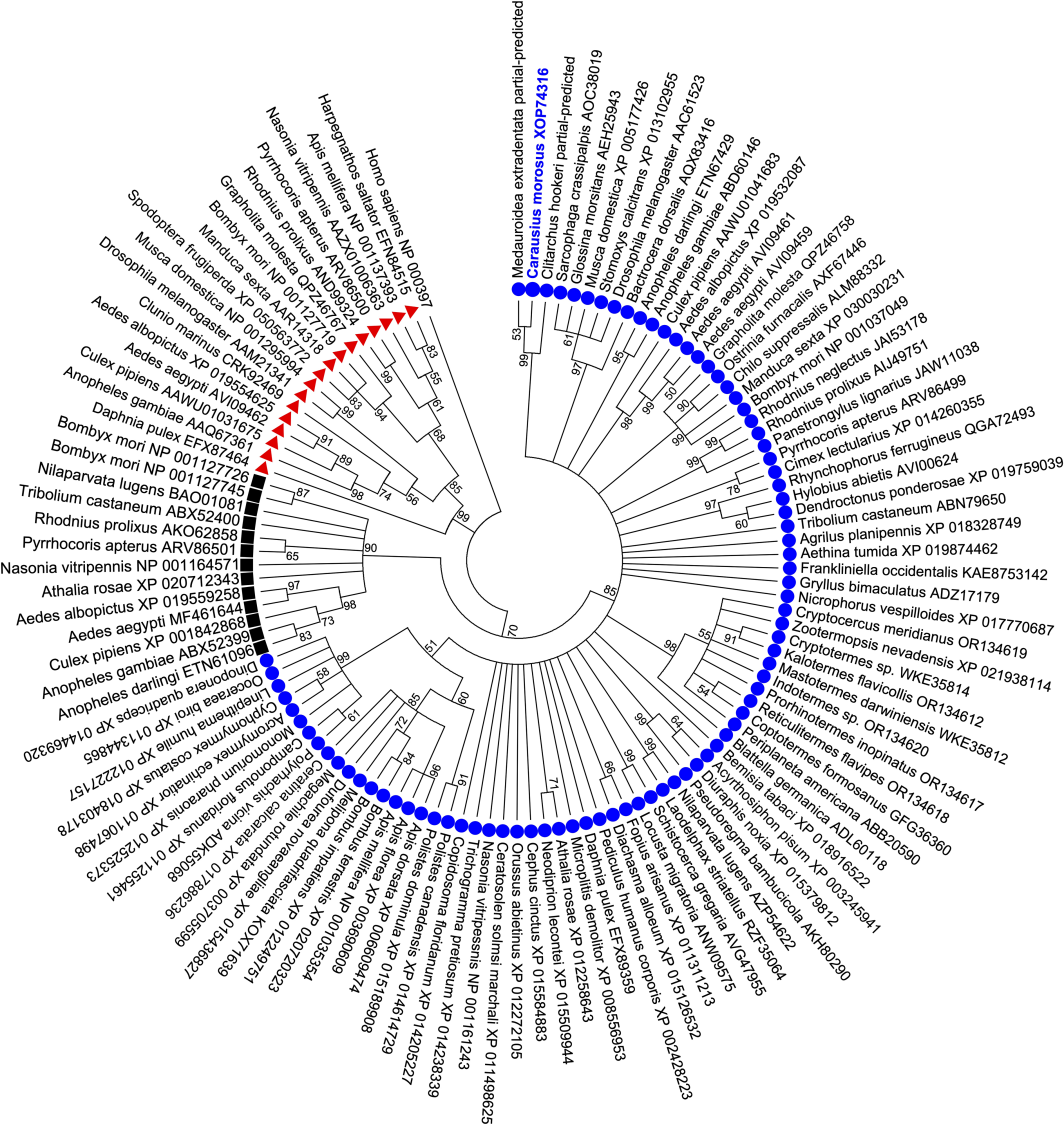
Phylogenetic analysis of *Carausius morosus* AKH receptor using maximum likelihood method, supports its classification as an arthropod AKH receptor. The bootstrap consensus tree is shown inferred from 1000 replicates representing the relationship of the three evolutionarily related GnRH-like peptide receptor families present in arthropods. Branches corresponding to partitions with less than 50% bootstrap support are collapsed. The *Carausius morosus* AKH receptor (Carmo-AKHR) is highlighted in blue text within the clade containing other AKH receptors (denoted by blue circles). The ACP receptors (denoted by black squares) are clustered in a sister clade to the AKH receptors while the Crz receptors (denoted by red triangles) cluster in a separate sister clade to the monophyletic group comprised of AKH and ACP receptors. Receptor protein sequences are labelled by species name from which they originate and their GenBank accession numbers. The human gonadotropin releasing hormone receptor isoform 1 (GenBank: NP000397) was included in the analysis and designated as the outgroup.

Phylogenetic analysis of the *C. morosus* AKH receptor protein sequence using the maximum-likelihood method was conducted to infer the relationship with other AKH receptors along with corazonin (Crz) and AKH/Crz-related peptide (ACP) receptors from other insects (**Figure 2**). The *C. morosus* AKH receptor characterized in the current study clusters within a clade with strong bootstrap support containing AKH receptors previously characterized or predicted based on genomic/transcriptomic data from other insects. The ACP receptors are positioned in a separate clade with high bootstrap support, which forms a sister group to the AKH receptors. Further, the monophyletic group containing the AKH and ACP receptor clades is a sister group to the clade containing Crz receptors (**Figure 2**), which is in agreement with previous studies describing these three evolutionary-related systems in arthropods including paralogous gonadotropin-releasing hormone (GnRH)-like peptides, namely AKH and ACP, along with Crz (44). Therefore, phylogenetic analysis of the relationship of the *C. morosus* AKH receptor with other insect AKH, ACP and Crz-type receptors resulted in a tree with highly supported topology containing three distinct clades for each receptor type and indicates the *C. morosus* receptor characterized herein is indeed an AKH receptor.

### 3.3 The identified receptor is a true adipokinetic hormone receptor

**Figure 3:**
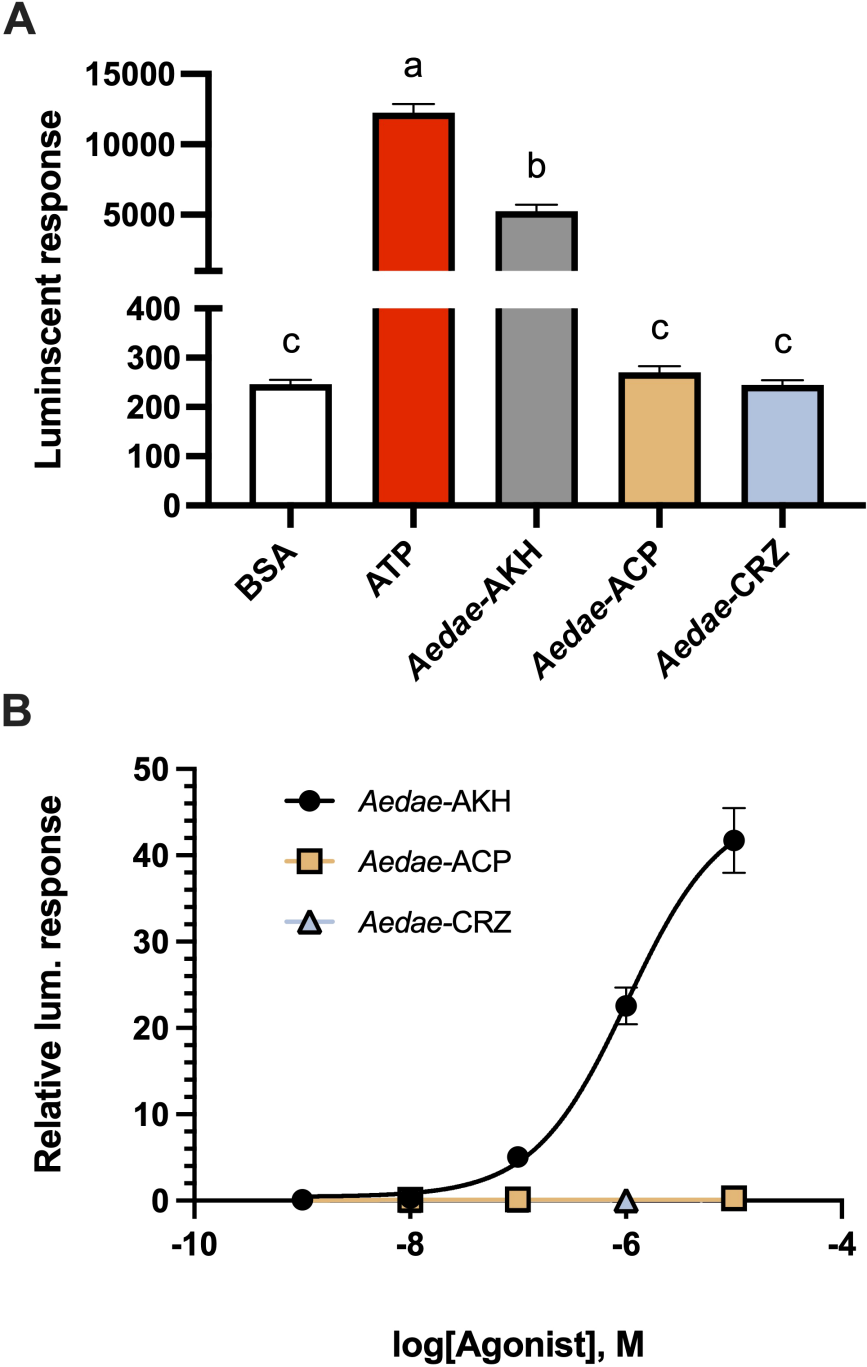
The identified receptor is a true adipokinetic hormone receptor with no sensitivity to other GnRH-related peptides found in insects. **(A)** Raw luminescent response to three GnRH-related peptide family members from *A*. *aegypti*, specifically Aedae-AKH, Aedae-ACP and Aedae-Crz tested at 10 µM concentration. Only Aedae-AKH was able to activate the *C*. *morosus* receptor demonstrating a significantly elevated luminescent response. **(B)** Dose-response analysis of three GnRH-related peptides from *A*. *aegypti* against the *C*. *morosus* AKH receptor with only Aedae-AKH resulting in dose-dependent receptor activation. In **(A)**, different letters denote bars that are significantly different from one another as determined by one-way ANOVA and Tukey’s *post-hoc* test; in **(B)**, data normalized to the maximum luminescent response using ATP (mean +/- SEM, n = 3).

Functional testing of the cloned 528 residue *C. morosus* AKHR receptor in the heterologous assay led to some encouraging results with specific activation by an AKH peptide (Aedae-AKH) observed in CHO-K1 cells stably expressing aequorin and transfected with the *C. morosus* AKHR, but not in cells transfected with eGFP (**Supplementary Figure S4A**). Despite co-expressing the murine promiscuous G protein, the luminescent response was a modest signal relative to background with ~3 fold greater luminescence than assay media (BSA) alone. A stable cell line was, therefore, prepared in efforts to improve the signal to noise ratio using a receptor construct in pcDNA3.1^+^ expression vector, that included mammalian codon optimization and co-expression of eGFP to monitor transfection efficiency and facilitate the selection of stable cells expressing the *C. morosus* receptor. All the resulting recombinant clonal lines tested (in the absence of the murine promiscuous G protein) demonstrated luminescent responses specific to AKH application that was not observed when cells were treated with CCHamide1 (**Supplementary Figure S4B**). Hence, supporting the AKH receptor sequence shown in **Figure 1**, as the true Carmo-AKHR. Three GnRH-related peptides of the yellow fever mosquito *A. aegypti* were tested on the expressed receptor construct *in vitro*, viz. Aedae-AKH, Aedae-Crz and Aedae-ACP. The positive control with ATP resulted in a maximal luminescent response of about 12 000, whereas the BSA (negative control) was at 220 (**Figure 3A**). Aedae-AKH at 10 µM achieved a luminescent response of about 5000, while Aedae-ACP and Aedae-Crz (each at 10 µM) did not have a higher value than the BSA control and failed to activate the receptor at all tested doses (**Figure 3B**). A full dose-response curve was attained with Aedae-AKH (**Figure 3B**). This is the first time that Carmo-AKHR is pharmacologically characterized and reveals that the receptor can only be activated by an AKH.

### 3.4 Structure-activity analysis with the endogenous AKH family peptides and modified Carmo-HrTH-II ligands: *in vitro* receptor assays

**Figure 4:**
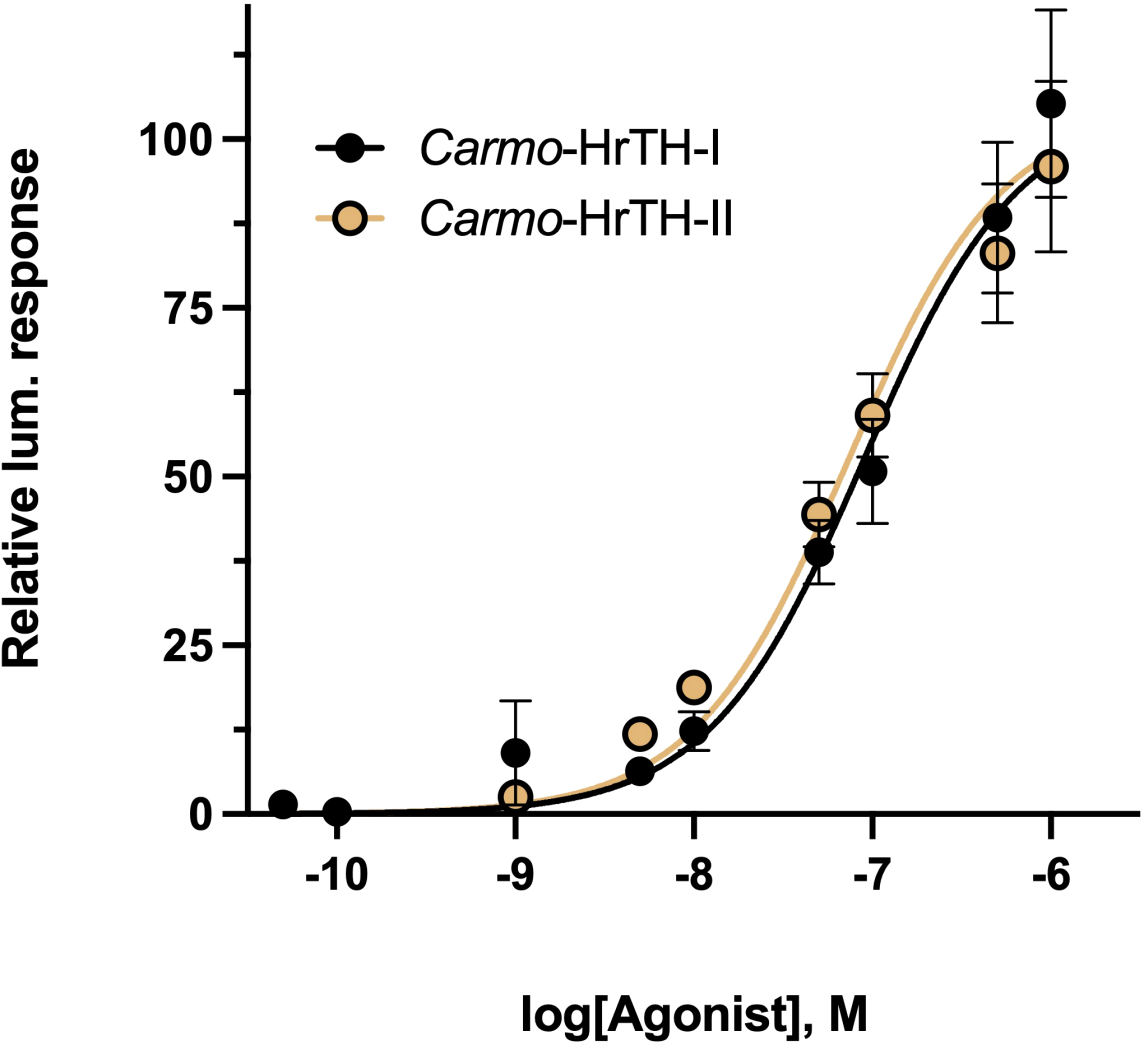
The effect of endogenous stick insect hypertrehalosemic peptides on stick insect AKH receptor activation. Dose-response analysis of two endogenous HrTH peptides, Carmo-HrTH-I and Carmo-HrTH-II, against the *C. morosus* AKH receptor. The primary structure of the endogenous HrTH peptides is identical, except that HrTH-I has an unusually modified Trp at position 8, with a C-bonded alpha-mannopyranose (see **Table 1**). Data normalized to the maximum luminescent response using ATP (mean +/- SEM, n = 3).

The two endogenous decapeptide hormones purified from *C. morosus* CC extract were tested for activating the expressed Carmo-AKHR. The full dose response curves of the two peptides, which differ only in a C-mannosylated Trp residue in compound I (**Table 1**), were nearly equipotent and resulted in roughly the same EC_50_ values for both endogenous peptides (**Figure 4A**).

**Figure 5:**
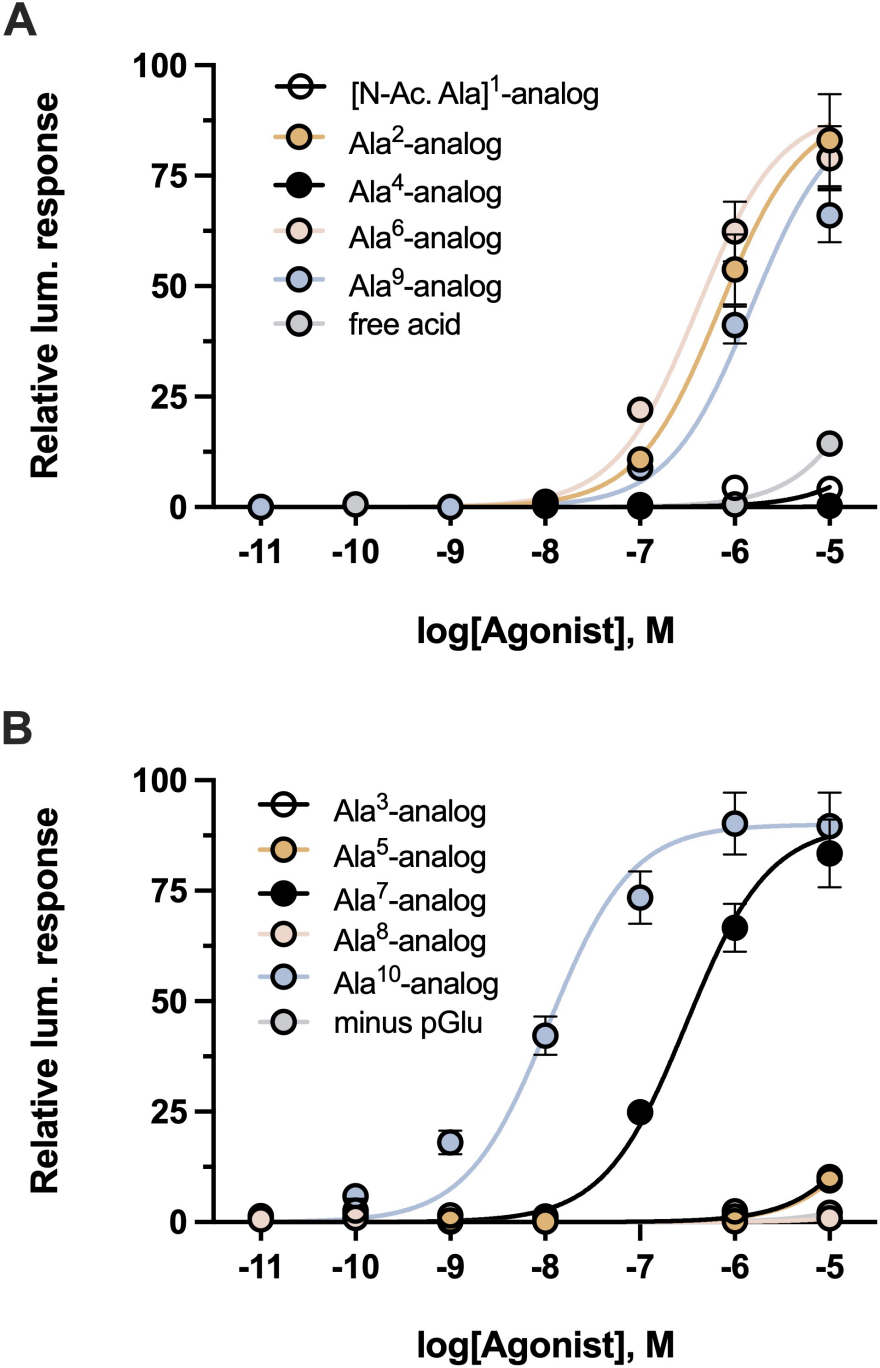
Activity of synthetic modified analogs of Carmo-HrTH-II on the stick insect AKH receptor. **(A)** Dose-response analysis of select synthetic analogs of Carmo-HrTH-II against the *C*. *morosus* AKH receptor, including alanine-substituted analogs in positions 1, 2, 4, 6 and 9 along with the non-amidated analog of Carmo-HrTH-II. **(B)** Dose-response analysis of distinct synthetic analogs of Carmo-HrTH-II against the *C*. *morosus* AKH receptor, including alanine-substituted analogs in positions 3, 5, 7, 8 and 10 along with an analog of Carmo-HrTH-II lacking the N-terminal pyroglutamic acid. Data normalized to the maximum luminescent response using ATP (mean +/- SEM, n = 3).

The influence of the peptide termini and the side chain of each of the 10 amino acids of Carmo-HrTH-II on the activation of Carmo-AKHR was tested by substituting each endogenous amino acid in the decapeptide with the simple amino acid Ala. The full dose response of receptor activation by each of these analogs is shown in **Figure 5** while **Table 1** summarizes the EC_50_ values obtained from the dose-response curves, and the peptide activity relative to Carmo-HrTH-II. If the blocked termini are replaced with N-Acetyl-Ala instead of pGlu at the N-terminus or a free acid is in place at the C-terminus instead of the amide, receptor activation is virtually absent, although a minimal but almost negligible activity can be shown with the highest concentration used of the free acid (**Figure 5A**).

Removing the N-terminal amino acid pGlu entirely, thus creating a nonapeptide, abolished activity (**Figure 5B**). Replacing the residues Thr at position 3 (**Figure 5B**) or Phe at position 4 (**Figure 5A**) or Trp at position 8 (**Figure 5B**) with Ala also failed to activate the AKH receptor of *C. morosus*, while a change of Thr at position 5 (**Figure 5B**) was also not well tolerated, resulting in a nearly 1200-fold reduction of activation (**Table 1**). Receptor activation was significantly less affected when Leu at position 2, Pro at position 6, Asn at position 7, or Gly at position 9, was replaced by Ala (**Figures 5A, B**; **Table 1**). The switch from Thr to Ala at position 10 (**Figure 5B**) resulted in receptor activation that rivaled that of Carmo-HrTH itself, with a calculated reduction of 0.15-fold (**Table 1**).

### 3.5 Receptor activation by naturally occurring AKH ligands and the effect of chain length

**Figure 6:**
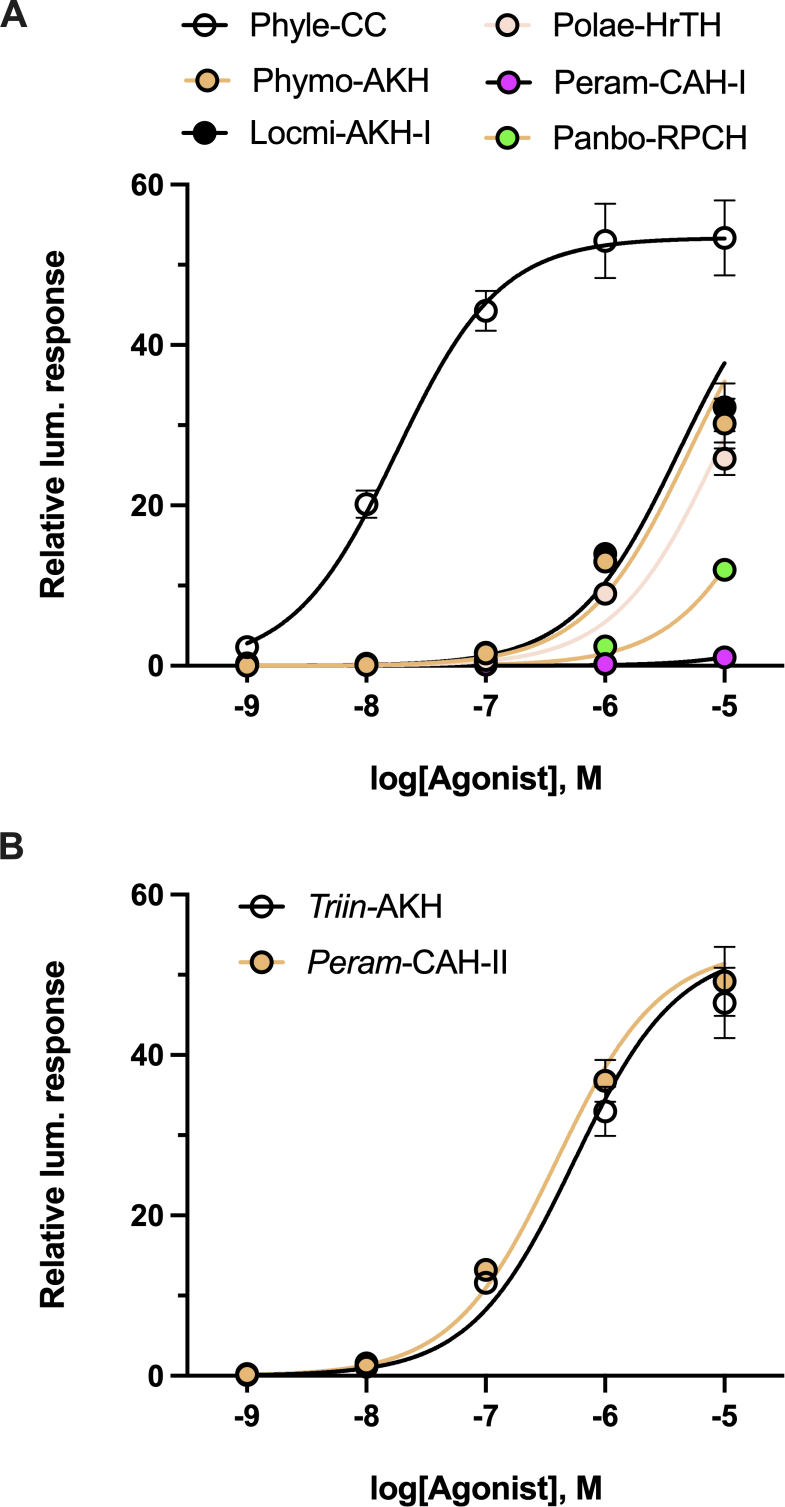
Activity of naturally occurring arthropod AKH analogs on the stick insect AKH receptor. **(A)** Natural AKH analogs with one, two or three non-alanine amino acid substitutions or different chain length compared to Carmo-HrTH-II. **(B)** Two naturally occurring AKH analogs from different insects (bioanalogs), Triin-AKH and Peram-CAH-II, which share the identical nine and eight, respectively, N-terminal residues to Carmo-HrTH-II and offer insights on the effect of shortened C-terminal chain length on the receptor selectivity for its endogenous ligands. Data normalized to the maximum luminescent response using ATP (mean +/- SEM, n = 3).

To try and understand the loss of ligand potency measured with some of the Ala-Carmo-HrTH analogs (see 3.4; **Table 1**), a selection of naturally occurring AKHs with one or two amino acid modifications (thus, a different side chain than the Ala substitution) and different chain length were tested in the *in vitro* receptor assay (**Figure 6**). Changing Thr at position 10 to Ser in a decapeptide as in Phyle-CC hardly made any difference with a trivial reduction of 0.24-fold (**Figure 6A**; **Table 2**). When the Thr at position 3 was changed to an Asn, as exemplified in the decapeptide Locmi-AKH-I, activity was reduced by over 1 order of magnitude or a reduction of more than 50-fold (**Figure 6A**). A further loss of potency (an almost 70-fold reduction in receptor activation) was observed when Asn in position 3 co-occurred with a Ser in position 10, as in the bioanalog called Phymo-AKH (**Table 2**). An octapeptide with an Ile at position 2 instead of Leu, such as in Polae-HrTH, was over two orders of magnitude less effective in activating the receptor with 120-fold reduced activity (**Table 2**; **Figure 6A**), whereas an otherwise identical octapeptide but with Leu in position 2, such as in Peram-CAH-II, had only a 5-fold reduction (**Table 2**; **Figure 6B**). Aedae-AKH, the octapeptide with a Ser residue at position 7 instead of an Asn, resulted in a 14-fold reduction in receptor activation (**Figure 3B**; **Table 2**). Octapeptides with multiple amino acid substitutions, as in Peram-CAH-I (Val^2^, Asn^3^ and Ser^5^) or in Panbo-RPCH (Asn^3^, Ser^5^ and Gly^7^) resulted in complete loss of efficacy in the former and a 455-fold reduction in the latter (**Figure 6A**; **Table 2**).

The nonapeptide Triin-AKH and the octapeptide Peram-CAH-II can be considered as C-terminally trunctated, amidated forms of Carmo-HrTH-II, thus, differing in chain length to the decapeptide Carmo-HrTH-II (**Table 2**). Full dose-response curves of this octa-and nonapeptide are depicted in **Figure 6B**. Both peptides activate the receptor well with EC_50_ values less than one order of magnitude higher than the endogenous decapeptides (**Table 2**).

## 4 Discussion

Sequence predictions had been made for the GPCR of the *C. morosus* AKH/HrTH peptides from two independently derived transcriptomes (3, 21). Here we report for the first time the full AKH GPCR sequence of *C. morosus* as deduced from PCR amplification, and with demonstrated functional activity. The reported sequence here differs somewhat from the published predictions: the two predicted AKH receptor variants (3) are very likely splice alternatives and shown in the current study to be partial sequences only; moreover, the true start codon of the complete AKH receptor is located 125 amino acids further upstream and is critical for receptor-ligand binding and function. The other prediction (21) referenced a transcriptome assembly and presented part of the receptor amino acid sequence only (position 187 to 479), where the C-terminus (residues 464 to 479) differed not only from the amplified AKH receptor of the current study, but also from the afore-mentioned published prediction of (3). This is enigmatic and likely due to an error on the part of those authors. Since the cloned receptor (528 a.a) is functional *in vitro*, there can be no doubt that the amplified receptor sequence reported here, is complete and correct.

The cloned receptor of the current study is specific for AKHs and does not recognize the related insect peptides of the GnRH superfamily, i.e. ACP and Crz, hence it is validated as Carmo-AKHR. Although this validation was performed with two peptides occurring in *A. aegypti*, this is of little consequence as the sequence of the ACP of *C. morosus* is identical to that in *A. aegypti*, and the Crz sequence of the stick insect differs only at position 7 (His instead of Arg) from the mosquito Crz. This finding aligns well with previous studies that have demonstrated no cross-activation between ligands and receptors between these three evolutionary-related signaling systems (26, 30, 32, 45).

Phylogenetic analysis indicates the *C. morosus* receptor has greatest similarity to other Phasmid AKH receptor sequences. Moreover, the receptors for GnRHR superfamily peptides in insects (AKH, ACP and Crz) are clustered within distinct clades, with strong statistical support (bootstrap percentages of 85% or higher in the current study). There is, however, not a consistently strong relationship of AKH receptors across insect orders. For example, the AKH receptors in Hymenoptera are not all clustered within a common highly supported clade; this may suggest differential evolutionary pressure driving receptor variation across species within this ancient order and may have implications for how different receptors interact with their cognate ligand or facilitate downstream signaling. An additional point is that, while the ACP receptor and its ligand are absent in the honeybee, *A. mellifera* (26, 45), this might be an exception in hymenopteran species given that we identified a candidate ACP receptor in the sawfly, *Athalia rosae* as well as in the parasitoid wasp, *Nasonia vitripennis* (30, 45).

Optimized *in vitro* receptor assays (involving heterologous expression of the Indian stick insect AKH receptor) were employed in the current study. Full activation of the Carmo-AKHR was achieved with both endogenous stick insect AKH/HrTH peptides, the Trp-mannosylated (Carmo-HrTH-I) and the non-mannosylated (Carmo-HrTH-II) isoforms with threshold at low nanomolar concentrations and in a dose-dependent fashion. The calculated EC_50_ value is almost equal, suggesting that the unusual post translational modification has no direct influence on receptor binding and activation. This agrees with measured biological data where no significant difference was obtained between the hypertrehalosemic activity of the two isoforms *in vivo* (14).

The receptor assay with Carmo-AKHR was also used to investigate the ligand structure-activity relationship, specifically examining the changes to receptor activation when the ligand termini were modified, or when the side chains of the various amino acids in the HrTH decapeptide were substituted with alanine or with different amino acids as found in naturally-occurring peptide isoforms from other arthropods (bioanalogs). Such studies had been carried out before with *in vivo* biological assays and ligated *C. morosus* specimens (16), thus, comparisons could be made from the direct method of assessing receptor activation and the indirect method with its many physiological and cellular complications. To date, this is a unique comparative opportunity where invertebrate AKH signaling (start and end point) is concerned.

### 4.1 The relevance of the AKH termini in activating Carmo-AKHR

The current study confirms the importance of the N-terminal pGlu and C-terminal amide of Carmo-HrTH-II: the terminally modified analogs showed no receptor activation. This absolute loss of activation is nonetheless surprising since a slight hypertrehalosemic activity (30%) was recorded with *in vivo* assays in *C. morosus* (16) and in *in vitro* receptor studies with *Drosophila melanogaster*, *Anopheles gambiae* and *Glossina morsitans morsitans*, the [N-Ac-Ala^1^] analog elicited approximately 20% to 35% receptor activity and the non-amidated AKH analog also activated the dipteran AKHR [40-70% activation (18, 19)]. In most *in vivo* studies of AKH ligand-receptor response, such as in the cockroach *Periplaneta americana*, the locust *Locusta migratoria* and the moth *Manduca sexta*, the AKHR could also still be activated by AKH analogs with other N-terminal blocked amino acids, such as [N-Ac-Ala^1^], [N-Ac-Tyr^1^], [N-Ac-Pro^1^], [N-Ac-Gly^1^], [biotin-Gly^1^] and even activated by peptides where Glu^1^ or Gln^1^ were not blocked and spontaneously underwent cyclization to pGlu [mediated by glutaminyl cyclases (46, 47)], and consequently resulted in a metabolic response, albeit quite lower than the native AKH/HrTH (48–51). In only two previously examined cases, the cockroach *Blaberus discoidalis* and the moth *Hippotion eson*, was physiological activity with [N-Ac-Ala^1^] or [N-Ac-Glu^1^] very minimal or completely abolished (52, 53). The lack of receptor activation (current study) with an unprotected nonapeptide in which the N-terminal pGlu residue was eliminated is in direct agreement with *in vivo* studies on stick insects (16), and results recorded with des-pGlu-Peram-CAH-I and -II in cockroaches and migratory locusts (54). The overall data sets suggest that the conformation of the N-terminally modified analogs differs considerably from the native conformation with pGlu, thus preventing proper binding to the AKH receptor and further, that the N-terminus is important to facilitate the correct peptide folding necessary for activating the AKH receptor. The interaction of pGlu with the AKH receptor has been shown to occur in the computational model for AKH ligand-receptor binding in the desert locust, *Schistocerca gregaria* and the waterflea, *Daphnia pulex* (55, 56). In different insects *in vivo*, the absence of a blocked C-terminus could result in no or poor cellular outputs (48, 53, 54), or this change was less critical for cell signaling (50). The *in silico* receptor modeling study of Birgül Iyison et al. (21) reported that the C-terminal part of Carmo-HrTH was surrounded by the binding pocket of the receptor. The ligand-receptor model of the waterflea also suggests that the RPCH C-terminus interacts with the AKH/RPCH receptor (55).

### 4.2 AKH chain length and Carmo-AKHR

*C. morusus* produces decapeptide AKHs that function optimally via the Carmo-AKHR. Two shorter bioanalogs with high identity to Carmo-HrTH were available for testing in the receptor activation assay: Triin-AKH, an amidated nonapeptide that ends in Gly^9^ and the amidated octapeptide, Peram-CAH-II that ends in Trp^8^. These peptides produced receptor activation curves with EC_50_ values similar to those of the endogenous AKH peptides (**Table 2**). Taken on its own, this may indicate that the C-terminal amino acids of Carmo-HrTH (thus Thr^10^ and Gly^9^) are not critical for activation of Carmo-AKHR, and that ligand length is not important for Carmo-AKHR. The *in vivo* bioassay data from previous studies with these peptides support the current findings of the Carmo-AKHR assays in part, for although Peram-CAH-II increased the concentration of circulating carbohydrates in the stick insect, the increase was only about a third of what the native decapeptide achieved (16). A similar biological response was also evident for the AKH receptor of the cockroach *B. discoidalis* where octapeptides were less than 30-fold active in increasing the carbohydrate titre compared to the endogenous decapeptide Bladi-HrTH (57). In these species, thus, the octapeptides show the same potency as the decapeptides but not the same efficacy (intrinsic activity) once the octapeptide was bound. In *S. gregaria* with two octapeptides and one decapeptide as endogenous AKHs, chain length does not play a role in receptor activation and biological activity, and all three ligands occupy the same binding site in the Schgr-AKH receptor (56). These data demonstrate once more that some AKH receptors are more promiscuous whereas others are strict in the characteristics of their ligand (26). The *C. morosus* AKH receptor clearly belongs to the latter category as demonstrated by the current pharmacological studies.

### 4.3 The iconic aromatic amino acids and an alternating hydrophobicity pattern are non-negotiable AKH features for binding Carmo-AKHR

Replacing the aromatic amino acids, Phe^4^ or Trp^8^ that are iconic for most natural members of the AKH family, with a nonpolar amino acid like Ala, completely abolished receptor activation in the current *in vitro* study. Earlier *in vivo* investigations on *L. migratoria* (58), as well as AKH/RPCH ligand-receptor model studies on *S. gregaria* and *D. pulex* made it clear that these two aromatic residues are critical for receptor binding (55, 56). To our knowledge there is no *in vitro* or *in vivo* SAR study where the substitution of these residues resulted in any biological activity or receptor activation, thus they are essential (see, for example, 18, 19, 48, 53). A very severe loss of activity was evident when replacing hydroxylated Thr^3^ or Thr^5^ with Ala in the current study. It was previously suggested that such substitutions disrupt the alternating hydrophilic-hydrophobic amino acid pattern of the molecule and that these N-terminal residues are involved in a beta-strand conformation (48) which is essential for receptor activation; Ala substitution studies in dipteran species have corroborated this notion (18, 19). In the *in vivo* study of the stick insect (16) all these analogs (Ala^3^, Ala^4^, Ala^5^ and Ala^8^) elicited no hypertrehalosemic effect and add to the credibility of the current receptor assay results.

### 4.4 Other ligand modifications and the effect on Carmo-AKHR activation

Replacing the Thr^10^ residue of Carmo-HrTH with Ala^10^ did not cause a reduction in receptor activation (current *in vitro* study) and a relatively small reduction in biological output was recorded *in vivo* (16), in agreement with *in vivo* bioassay in *L. migratoria* (59) and *B. discoidalis* (52). The AKH isoform of the grasshopper *Phymateus leprosus*, Phyle-CC, that differs from Carmo-HrTH-II only by the substitution of a hydrophilic Ser^10^ for the usual hydrophilic Thr^10^ is also not much (*in vitro*) or not at all (*in vivo*) affected in its action. Clearly, this position in the AKH ligand appears not to be essential for Carmo-AKHR activation and for biological activity, in contrast to the octapeptide in **4.2** where potency is affected. However, this contrasts with the modeling report that implied the importance of the entire C-terminal part of Carmo-HrTH for binding to its receptor (21). Thr^3^ on the other hand is clearly an important amino acid residue in the functioning of Carmo-HrTH for when it is substituted by Ala or by a hydrophilic Asn (which has a carboxyamide sidechain) - as realized in the decapeptide Locmi-AKH-I, receptor activation is reduced 56-fold (**Table 2**) and biological function is impaired (16). Similarly, Phymo-AKH that is produced in the grasshopper *Phymateus morbillosus* with Thr^3^ and Ser^10^ resulted in severely reduced receptor activation *in vitro* (almost 70-fold, current study) and absence of function *in vivo* (16). All these data corroborate the Ala replacement results and point to a clear preference for Thr in position three of Carmo-HrTHs and its importance for the receptor binding process. It is also interesting to note that the AKH receptor in the moth *H. eson* has a lower affinity for Asn^3^ instead of the usual Thr^3^ (53), whereas Asn^3^ is preferred for the *L. migratoria* AKH receptor *in vivo*, thus showcasing yet again that co-evolution of ligand and receptor is one of the driving forces in achieving selectivity.

The importance of position 2 in Carmo-HrTH is quite clear: the current *in vitro* receptor study show only a small reduction in receptor activation when Leu^2^ with its bulky alkyl side chain was replaced by the simple Ala^2^ (-CH_3_) side chain and the earlier *in vivo* data report the same increase of circulating carbohydrates as with the parent molecule (16). Again, this is reminiscent of the situation in *B. discoidalis* where Ala^2^ substitution for Val^2^ is well tolerated (52). In the current study we found that the octapeptide Peram-CAH-II with a Leu^2^ had only a 5-fold reduction in receptor activation; if, however, Leu^2^ was replaced with the stereoisomer (β-branched) Ile^2^ - as in the natural peptide Polae-HrTH of the cockroach *Polyphaga aegyptiaca*, the reduction was increased to 120-fold. Thereby suggesting that the orientation of the alkyl side chain made a difference and that it was essential to present the ligand to the receptor in a very specific orientation. Previously it had been reported, for example, for the AKH receptor of *H. eson* and the RPCH receptor of the shrimp *Palaemon pacificus* by *in vivo* experiments that substitution of the natural Leu^2^ by Ile^2^ achieved less activity but Val^2^ as a replacement was even less active (53, 60). In conclusion, physical differences of side chains in the ligand may be crucial for correct docking of the ligand to its receptor.

The secondary structure of various members of the AKH family are predicted by NMR spectroscopy to have a beta-bend structure, and this is especially pronounced in those peptides containing Pro^6^ (see, for example, 56, 61, 62). Such “turns” are confined to amino acids in positions 5 to 8. Carmo-HrTH-II, the lead peptide in the *in vivo* studies (16) and in the current *in vitro* receptor assays, has a Pro in position 6; NMR experiments with Carmo-HrTH-II to assign its secondary structure should clarify the conformation of the molecule with a beta-bend structure, or otherwise. Biological assay data support the presence of a turn in Carmo-HrTH-II as no or very little hypertrehalosemic activity was obtained with Ala replacements at positions 5 to 8 (16), i.e. when a β-bend could not be formed. The current *in vitro* work supports this notion in part: residues 5 and 8 are essential for receptor activation (as discussed above; **Table 1**) but the substitution of residues 6 or 7 with Ala did not abolish receptor activation (with not more than a 5.5-fold reduction in activation relative to the endogenous peptide, Carmo-HrTH-II; **Table 1**). This is reminiscent of receptor assay studies on AKH receptors in Diptera (18, 19). In addition, biological assays with cockroaches also demonstrated that the substitution of the 7^th^ amino acid, apparently, does not hinder the interaction of the peptide with its receptor through the usual backbone hydrogen bonding (48, 52).

In conclusion, the combined data sets from the current *in vitro* receptor activation assays and earlier *in vivo* bioassays with *C. morosus* have produced a comprehensive view into the ligand requirements for activating the cloned Carmo-AKHR and for producing a biological result. The veracity of the cloned receptor sequence and the importance of the conserved AKH features are, thus, substantiated. Nevertheless, a minority of tested analogs produced contradictory results in the two assay systems, which in itself is not entirely surprising but shows that additional information must be sought via other experimental means to have a complete picture of AKH signaling in the Indian stick insect. *In vivo* assay results are prone to the influence of various (uncontrollable) factors, such as degradation of the injected ligand by peptidases; the release of another peptide(s) in the live insect with unknown effects; the possibility of an unknown promiscuous receptor that may bind the ligand; and/or feedback pathways that are activated/inhibited to ameliorate the cellular output. The *in vitro* receptor assay, on the other hand, specifically eliminates these biological elements and focuses only on receptor activation as determined in the current study by a bioluminescent response arising from calcium-dependent activation of the photoprotein aequorin in the cell construct. While there is broad evidence supporting AKH receptors induce calcium signaling both from *in vivo* (63–65) and *in vitro* (26, 32, 66, 67) studies, there is also evidence for the involvement of other second messengers (see 68). To our knowledge, the *in vivo* signal transduction for endogenous *C. morosus* AKH peptides has not been investigated, but the *in vitro* data from the current study provides evidence supporting the involvement of calcium signaling. Most importantly, the receptor assay cannot give insight into the subsequent steps or events in receptor signaling (the signal transduction pathway). Some of the contradictory results from our receptor assay and bioassay (e.g. with Ala^6^, Ala^7^ and Ala^10^ analogs, or with octapeptide bioanalogs) clearly points to other dynamics at play beyond the mere activation of the Carmo-AKHR. We propose that studies utilizing distinct heterologous assays to measure levels of other second messengers, such as cyclic AMP involving stimulatory (Gs) or inhibitory (Gi) G proteins could provide insight on the native signaling cascades necessary for AKH-AKHR signaling in the Indian stick insect, while the construction of a ligand-receptor binding model via NMR and molecular dynamics, can provide additional insights into how the ligand interacts with Carmo-AKHR by calculating binding energy. Although ligand binding *per se* does not mean activation of the transduction cascade in each case, as has been outlined before (69), it may shed light on the binding dynamics and differing molecular interactions of peptide analogs to explain the anomalous results from biological assays and receptor assays.

Finally, can the characterized Carmo-AKHR shed any light on the phenomenon that an AKH effect *in vivo* is only achieved after a ligature is applied to separate circulation between the head of the stick insect and the rest of its body? This phenomenon, together with the observation that octopamine (the equivalent of adrenaline in insects) resulted in slowing the heartbeat of *C. morosus*, led us to conclude that this is by design and fits with the low energetic, anti-predation behavioral strategy of stick insects, viz. thanatosis (15, 16). To succeed in playing dead, the musculature of *C. morosus* must contract/relax rapidly to resemble the surrounding twigs of its environment; the findings that myoinhibitory peptide from the common inhibitor and dorsal unpaired median neuron groups, is the only neuronal peptide modulator of all the leg muscles in *C. morosus* and that the excitatory motor neurons in the leg muscles contained no neuropeptides (3) – in contrast to other animals that engage in fight or flight -is therefore additional support for how the stick insect can successfully engage predator avoidance mode. Can one glean the underlying mechanism (e.g. an inhibitory G-protein, and/or a substance released from the head that ultimately blocks the AKH effect) from the *in vitro* expression system? Our heterologous receptor assay system did not rely on Gi signaling, but Gq-mediated calcium mobilization. Thus, while *in silico* prediction (70) of Carmo-AKHR G protein specificity suggests Gi coupling might be involved, we have no empirical data to support it. Furthermore, Gi coupling of Carmo-AKHR may explain the lack of metabolic response observed *in vivo* to Carmo-HrTH when injected in unligated stick insects, but it does not explain the measurable metabolic response in ligated stick insects and points rather to a mechanism involving a second substance/pathway emanating from the head that can be bypassed by blocking the circulatory system from the head. Presumably, in the ligatured stick insects, the unknown substance (possibly an antagonist or a factor causing the release of an antagonist) does not reach the fat body, where the AKHR is enriched; Carmo-HrTH can then bind the AKHR and elicit the classic hypertrehalosemic response *in vivo*. Clearly, this is an exception compared to other insects. One outlook may be to use fat body tissue from *C. morosus* in *in vitro* tests with AKHs and other substances, to provide insight into whether AKH activity *in vivo* is being inhibited by a brain-derived factor that is blocked during neck ligature, thus allowing AKH to elicit its energy mobilizing activity. In a pioneering study on endogenous AKH peptides and physiological effects on metabolism in *C. morosus*, it was clearly demonstrated that glycogen phosphorylase from the stick insect fat body showed significantly increased activity following injection of a crude extract of corpora cardiaca in ligated as well as in unligated stick insects (7). The authors of that study argued against an inhibitory factor that denied trehalosemic activity in unligated insects, and argued for an intriguingly simpler physical explanation, instead. Namely, that the Indian stick insect contained only low levels of glycogen in the fat body with a consequent tiny increase in released trehalose; this small increase, however, became more “visible” in the reduced volume of hemolymph in a ligatured insect (7).

## Data Availability

The datasets presented in this study can be found in online repositories. The names of the repository/repositories and accession number(s) can be found in the article/**Supplementary Material**.
