# Supplementary material for: Sequence, characterization and pharmacological analyses of the adipokinetic hormone receptor in the stick insect, Carausius morosus
Source: Front Endocrinol (Lausanne). 2025 Jul 17;16:1601334. doi: 10.3389/fendo.2025.1601334 (PMC12310481; doi:10.3389/fendo.2025.1601334)
Supplement: Supplementary file 1 [file DataSheet1.pdf]

Supplementary Material

Sequence, characterization and pharmacological analyses of the adipokinetic hormone receptor in the stick insect, *Carausius morosus*

Gerd Gäde[1*], Jinghan Tan[2], Salwa Afifi[2], Jean-Paul Paluzzi[2], Graham Jackson[3], Heather G. Marco[1]

[1] Department of Biological Sciences, University of Cape Town, South Africa
[2] Department of Biology, York University, Toronto, ON, Canada
[3] Department of Chemistry, University of Cape Town, South Africa

*Corresponding author: gerd.gade@uct.ac.za

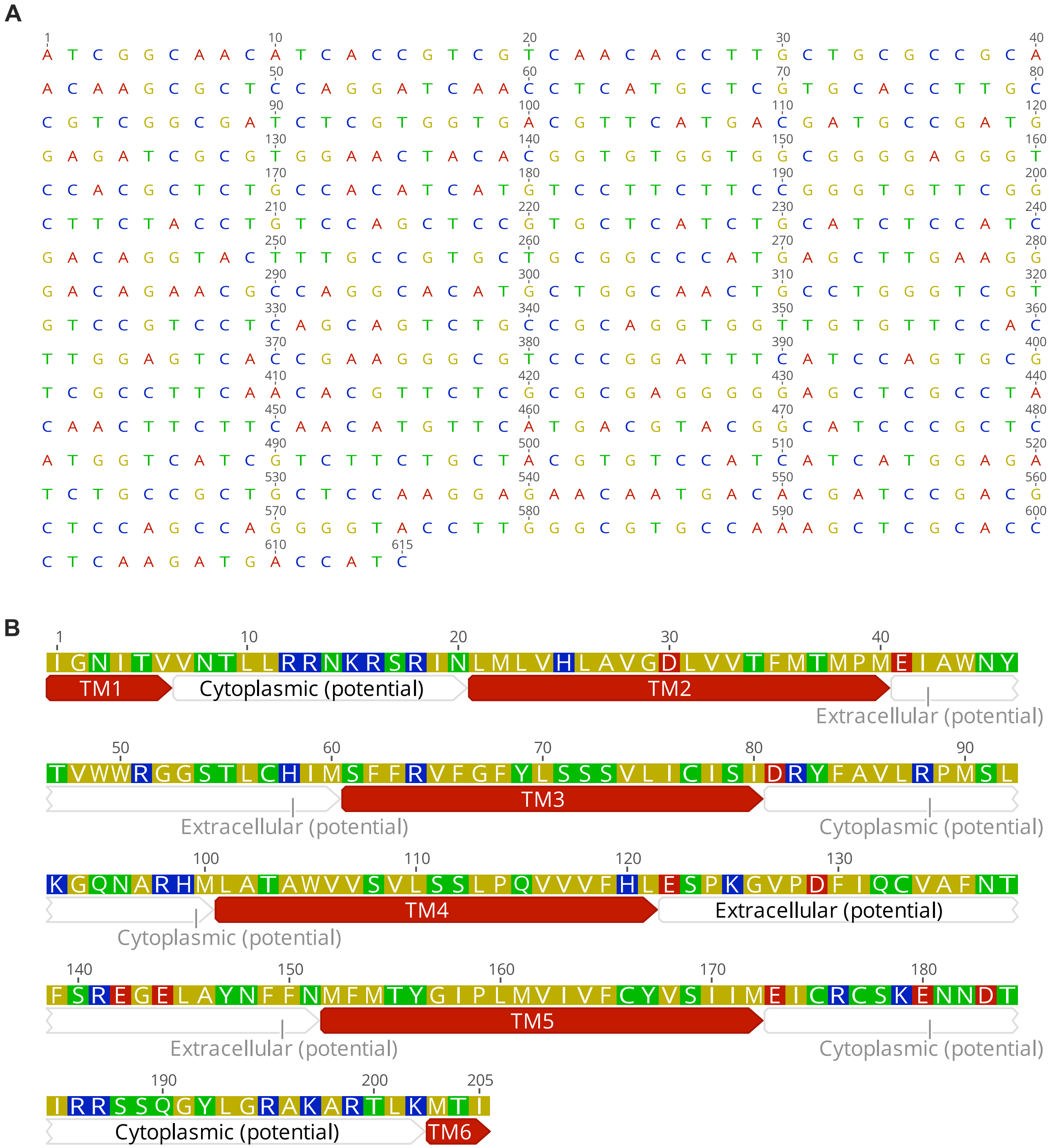

Supplementary Figure 1. The partial 615 bp nucleotide (A) and deduced 205 residue protein sequence (B) of the *Carausius morosus* AKH receptor (Carmo-AKHR) obtained using degenerate primers that were designed based on hits within available Phasmid genomes (*Clitarchus hookeri* and *Medauroidea extradentata*) screened by tblastn analysis with the desert locust AKHR protein sequence (GenBank accession# AVG47955) as a query.

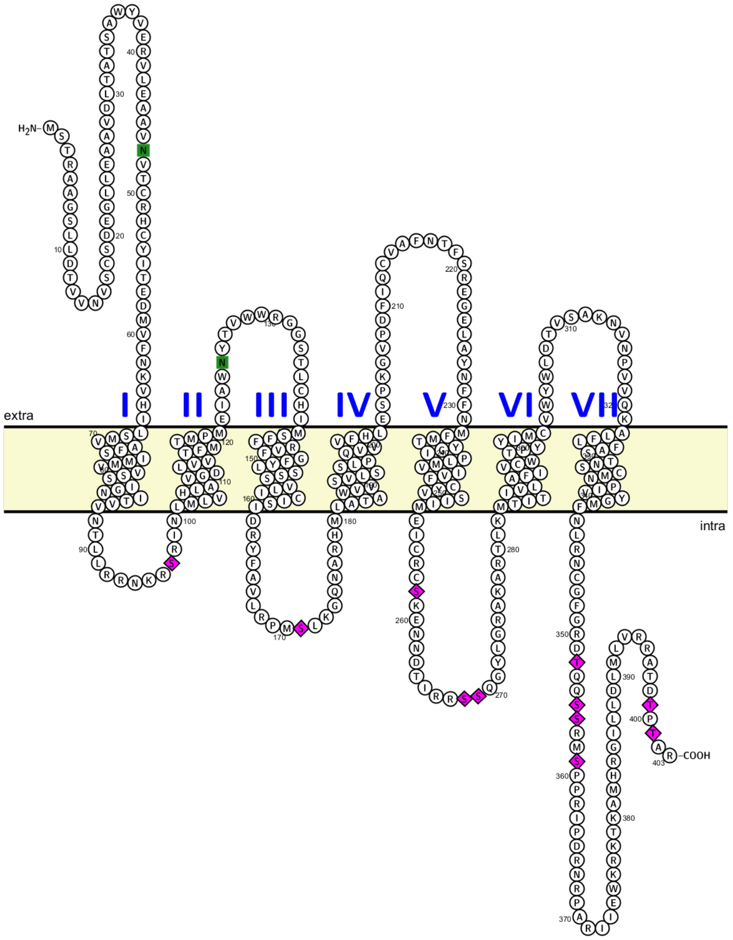

Supplementary Figure 2. Predicted topology and post-translational modifications of the incomplete and non-functional *C. morosus* AKH receptor. The deduced 403 amino acid Carmo-AKHR protein sequence was used to predict membrane topology (residues in membrane), N-linked glycosylation sites (residues denoted by green squares) along with potential phosphorylation sites within cytoplasmic domains of the receptor (residues denoted by purple diamonds). See the methods section for full description of steps in the analysis and prediction of post-translational modifications along with references for the web-based applications. Protter, an open-source tool, was used prepare the schematic of the receptor topology as well as label the predicted post-translational modifications.

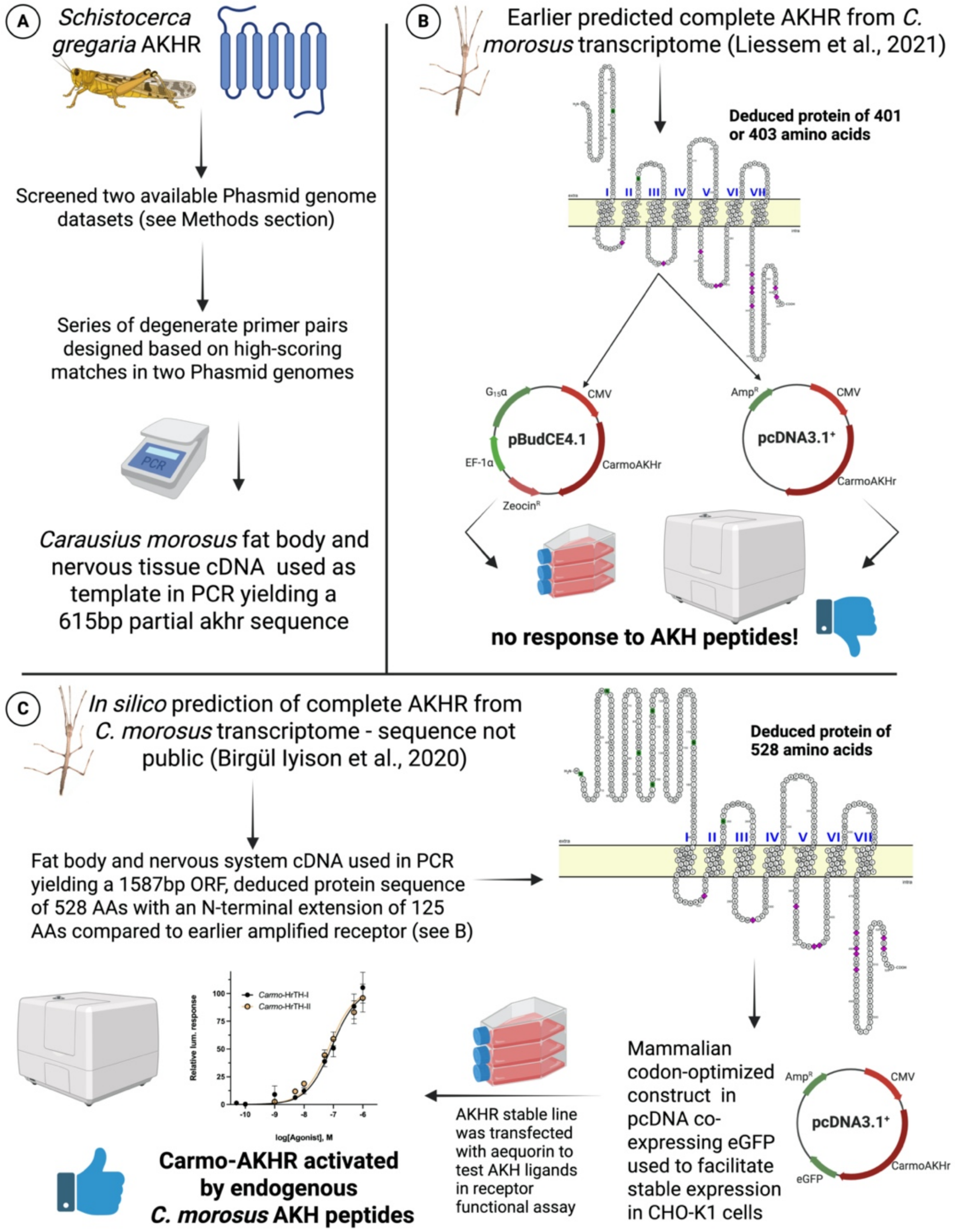

Supplementary Figure 3. Overview of cloning strategy to obtain the functional AKH receptor in *Carausius morosus* (Carmo-AKHR). A) Summary of initial amplification of partial Carmo-akhr sequence prior to genome or transcriptome data being available. The desert locust AKHR protein sequence was used to screen two stick insect genomes, for the design of degenerate primers. An incomplete (partial) receptor sequence was amplified from *C. morosus* cDNA templates, including fat body and nervous tissue. B) Summary of cloning strategy following reported transcriptome dataset including two AKH receptor isoforms (Liessem et al., 2021); neither of these receptor variants were found to be activated by AKH peptide ligands irrespective if expressed alone (using pcDNA3.1$^+$) or co-expressed with a promiscuous G protein (using pBudCE4.1 containing Galpha15, $G_{15}\alpha$). C) Summary of cloning strategy following discussion with the lead author of an earlier *in*

silico study that predicted and characterized *C. morosus* AKHR without having reported the full receptor sequence or depositing it in a public database (Birgül Iyison et al., 2020). In our study, a mammalian codon-optimized construct in pcDNA3.1^{+} was used, co-expressing eGFP to facilitate stable cell line selection in CHO-K1 cells. Several Carmo-AKHR stable lines were tested and found to be activated by a prototypical AKH peptide. A stable line with optimal signal-to-noise ratio was scaled up for use in multiple biological replicates testing endogenous AKH ligands as well as natural and synthetic analogs. Figure created in BioRender. Paluzzi, J. (2025) https://BioRender.com/s0l94lx

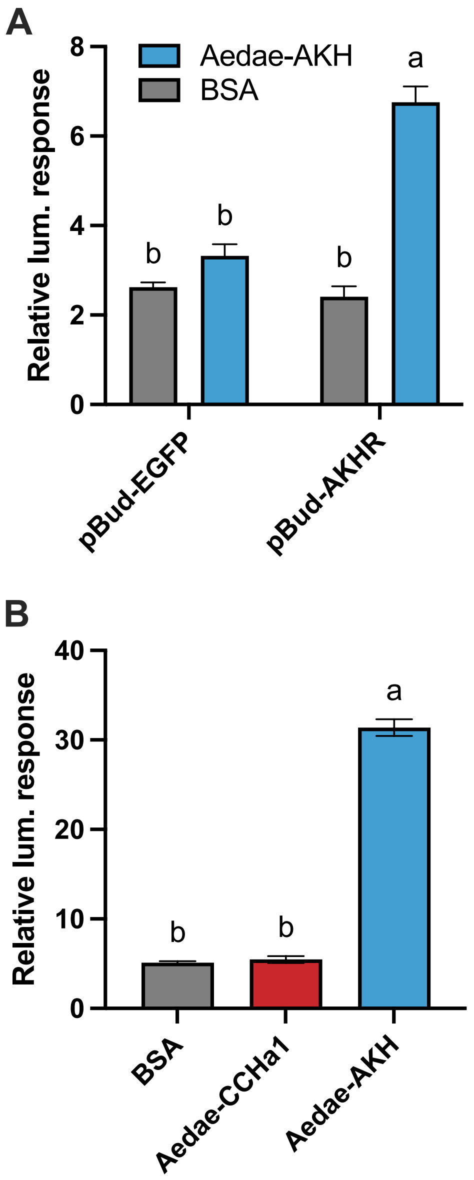

Supplementary Figure 4. Activity and specificity of a recombinant Carmo-AKHR in a heterologous *in vitro* receptor assay using transient expression of the cloned Carmo-AKHR in pBud co-expressing

promiscuous G protein (Galpha15) in CHO-K1 cells and stably expressing aequorin. Significant luminescence in response to an AKH peptide (Aedae-AKH) was observed only in cells expressing Carmo-AKHR but not in cells transfected with EGFP within the same expression plasmid, as determined by two-way ANOVA followed by Tukey's post-hoc test. (B) Clonal Carmo-AKHR recombinant CHO-K1 cell line using a mammalian codon optimized construct in pcDNA3.1^{+} expression vector. Shown is the recombinant cell line with the highest luminescent response and greatest signal-to-noise ratio which was specifically activated by Aedae-AKH while showing no response to a different neuropeptide family member (Aedae-CCHa1) as determined by one-way ANOVA and Tukey's post-hoc test. These specific peptides are known from the yellow fever mosquito *Aedes aegypti*. Data normalized to the maximum luminescent response using ATP (mean ± SEM, n = 3-4).
